# Minimal progress toward sustainment: 10-year replication of substance use EBP sustainment trajectories and associations with implementation characteristics

**DOI:** 10.1186/s13012-025-01471-2

**Published:** 2025-12-02

**Authors:** Alex R. Dopp, Michelle Bongard, Bing Han, Grace M. Hindmarch, Mekdes Shiferaw, Sapna J. Mendon-Plasek, Baji Tumendemberel, George Timmins, Kendal Reeder, Philip Pantoja, Danielle Schlang, Lora L. Passetti, Mark D. Godley, Sarah B. Hunter

**Affiliations:** 1 RAND, 1776 Main Street, Santa Monica, CA 90401 USA; 2 Department of Research and Evaluation, Division of Biostatistics Research, Kaiser Permanente Southern California, 100 South Los Robles Avenue 2nd Floor, Pasadena, CA 91101 USA; 3 Department of Health Policy and Management, University of California Los Angeles, 650 Charles Young Dr. S., 31-269 CHS, Box 951772, Los Angeles, CA 90095 USA; 4 Department of Psychology, University of California Los Angeles, 1285 Psychology Building, Box 951563, Los Angeles, CA 90095 USA; 5 Chestnut Health Systems, 1003 Martin Luther King Dr., Bloomington, IL 61701 USA

**Keywords:** Youth substance use, Substance use disorder treatment, Evidence-based practices, A-CRA, Financing strategies, Public finance, Policy, Implementation, Sustainment, Replication

## Abstract

**Background:**

Over the past decade, implementation researchers have empirically identified factors influencing long-term sustainment of evidence-based practices (EBPs) to target in implementation efforts. We examined progress toward promoting sustainment by conducting a conceptual replication of a prior study (Hunter et al., 2015, *Implementation Science*) that measured sustainment of an exemplar EBP for youth substance use, the Adolescent Community Reinforcement Approach (A-CRA).

**Method:**

Data were collected 1–5 years after initial implementation funding ended (*M* = 3.3 years) through interviews and surveys with clinicians and supervisors from service organizations that implemented A-CRA (*n* = 66). Using survival analysis, we calculated the probability of A-CRA sustainment (dichotomously reported [yes/no] in interviews) over time and examined associations with contextual factors across the multilevel domains of the Consolidated Framework for Implementation Research (CFIR). We also combined our data with Hunter et al. (*n* = 68) to test if sustainment status or interactions with contextual factors differed by sample, and used rapid qualitative analysis of interviews to further explore patterns in the quantitative findings.

**Results:**

In our sample, A-CRA sustainment probability decreased over time; 71% of organizations were sustaining A-CRA when funding ended, whereas only 33% were sustaining 5 years later; this survival curve did not statistically differ from Hunter et al. Sustainment was significantly associated with factors across CFIR domains: we replicated associations found by Hunter et al. (with e.g., funding stability, available clinicians, intervention complexity) and found unique associations (with e.g., program evaluation and strategic planning capacities, available supervisors, and perceived advantages and success of A-CRA). One association from the prior sample did not fully replicate (*p* < .10), but there were no significant interactions between contextual factors and sample. Qualitative findings further contextualized these results with service organization perspectives on factors influencing sustainment.

**Conclusions:**

Our findings suggest that work over the past decade promoting sustainment of EBPs for youth substance use may not have produced measurable impacts. Future work needs to better incorporate growing knowledge on sustainment predictors into development and testing of robust, multilevel implementation strategies and system-level supports. This study also provides a useful illustration of a replication study in implementation science, which are important but rare.

**Supplementary Information:**

The online version contains supplementary material available at 10.1186/s13012-025-01471-2.

Contributions to the literature

- Our analyses demonstrate equivalent sustainment trajectories for an EBP for youth substance use treatment in two distinct samples collected a decade apart, suggesting that work over the past decade promoting sustainment has not necessarily produced measurable impacts.
- Replication of associations between a wide range of multilevel factors and probability of sustainment suggest that the drivers of sustainment challenges have also remained largely consistent – offering high-priority targets for implementation strategies and supports.
- Given that replications in implementation science are important, yet remain rare, this study provides a useful illustration of how implementation researchers can approach replication of other studies.

## Background

The past decade has seen significant advances in research on sustaining evidence-based practices (EBPs), defined as long-term practice delivery that maintains recipient benefits [1]. Studies consistently document sustainment rates of ≤ 50% within ~ 2 years across varied EBPs, contexts, and implementation strategies [2, 3], suggesting considerable need for improvement in supporting sustainment. Major frameworks describing multilevel contextual influences on implementation now attend to key drivers of sustainment [4–6]. For instance, the Integrated Sustainability Framework [6] synthesizes how characteristics of settings (e.g., funding/resources), EBPs (e.g., complexity), and implementation processes (e.g., provider factors) collectively influence sustainability. Empirical studies [7, 8] have shown how these drivers can differ from drivers of initial implementation. However, it is unclear to what extent our increased knowledge of sustainment processes has improved sustainment outcomes in practice settings. The present study examined progress toward promoting sustainment by replicating an early study, published 10 years prior, and comparing the findings between studies.

Replication refers to systematic efforts to test whether two or more studies produce the same finding (within the limits of sampling error) [9]. Replication studies are increasingly emphasized in social sciences as a key foundation of rigorous, reliable knowledge. However, replications remain rare in implementation science [10], a field in which direct replication is challenging because the findings of each study are influenced by study-specific combinations of multilevel contextual influences. *Conceptual* replications [11, 12] – which examine generalizability of findings across variation in key contextual factors such as setting, EBP, or target population – may be especially valuable for advancing implementation science [10].

We conceptually replicated a study by Hunter et al. [13] that examined sustainment of an exemplar EBP for youth substance use, the Adolescent Community Reinforcement Approach (A-CRA) [14], in treatment organizations that received federal grants to implement A-CRA (i.e., “organization-focused” grants). They found that probability of A-CRA sustainment rapidly decreased over the first two years after grant funding ended, and that sustainment was associated with multilevel contextual factors (e.g., funding stability, organizational focus on substance use services, available clinicians, provider-rated A-CRA complexity). We replicated Hunter et al.’s approach with more recent cohorts of treatment organizations that implemented A-CRA through federal grants awarded to state substance use service agencies (i.e., “state-focused” grants). Our research questions were (1) Do quantitative findings from Hunter et al. replicate in state-focused grantee cohorts? and (2) How do qualitative perspectives of A-CRA providers aid in understanding findings across samples?

## Method

We describe this study using reporting guidelines for observational studies [15] (see Additional File 1). All procedures were approved by the RAND IRB (Protocol #2020-N0887).

This replication was part of ongoing research comparing A-CRA implementation outcomes between organization-focused versus state-focused grants; see our protocol [16] for details of the grant initiatives (funded by the U.S. Substance Abuse and Mental Health Services Administration, SAMHSA) and project. We provide an overview of the state-focused sample and replication analysis methods here. The original study (Hunter et al.) [13] is published; we summarize its sample, methods, and results in Additional File 2 for reference, because (a) we replicated that study’s recruitment strategies, data sources, measures, and analyses; and (b) we included the original sample in certain replication analyses to enable direct comparisons.

### Sample and recruitment

#### Treatment organizations

The replication sample included 88 treatment organizations from 18 states that received state-focused grants between 2012–2021. SAMHSA intended for state agencies to (a) develop A-CRA-focused funding and training infrastructure (to improve statewide conditions for A-CRA sustainment); and (b) partner with treatment organizations to implement and sustain A-CRA (like organization-focused grants).

#### Staff

We recruited providers from treatment organizations involved in state-focused grants via phone and email. Data collection took place within 5 years of grant end (December 2021–June 2022). We aimed to include both clinicians and clinical supervisors delivering youth substance use treatment at each organization; those knowledgeable about A-CRA were preferred. We obtained informed consent, collected de-identified data only, and compensated providers $25 USD per research activity. Of 88 eligible organizations, 66 had staff participate in the interview and survey (75% response rate).

### Data sources

#### Interviews

Semi-structured interviews were conducted via phone and used a combination of open-ended questions and standardized probes, tailored to the provider’s role and whether the organization still delivered A-CRA (see Additional File 3 for protocols). Interviews were audio-recorded and transcribed, and lasted ~ 45–60 min.

#### Provider surveys

Following each interview, providers were sent a web-based survey that collected standardized measures and other descriptive information (see Additional File 4 for survey items). Completion averaged ~ 25 min.

### Measures

#### A-CRA sustainment status

In interviews, we asked whether the organization currently delivered A-CRA (yes/no) and verified the grant end date. For organizations not sustaining A-CRA, we collected the date A-CRA delivery stopped in the follow-up survey. We calculated the time in months from grant end to the latest interview date and, if relevant, A-CRA stop date for each organization.

#### Contextual factors

Informed by the Consolidated Framework for Implementation Research (CFIR), our survey assessed multilevel factors predicted to influence sustainment. Interviews also explored providers’ perceptions of multilevel barriers and facilitators to A-CRA sustainment.

*Setting characteristics* included organizational focus (i.e., whether the primary focus of services was substance use), number of services offered (from a list of 17 categories), and eight EBP sustainment capacity domains measured by the Program Sustainability Assessment Tool (PSAT) [17, 18].* Implementation characteristics* were the number of clinicians and clinical supervisors employed at grant end at each organization who completed all A-CRA training requirements (i.e., were certified in A-CRA). *Intervention characteristics* included provider ratings of A-CRA complexity [19], relative advantage [19], implementation difficulty [20], and perceived success [20]. When multiple participants from an organization provided ratings, we averaged those ratings.

### Analytic plan

We used a sequential, primarily quantitative mixed-method approach (QUAN **→** qual) [21]. First, we conducted quantitative analysis that (a) replicated Hunter et al. [13] with the state-focused sample and (b) tested for differences by sample in a combined model. Second, we explored qualitative data from both samples that provided context for the observed quantitative results.

#### Survival analysis (QUAN)

First, we calculated the Kaplan–Meier survival probability curve representing the probability that an organization sustained A-CRA across five years post-grant. Sustainment outcomes were right censored by (i.e., could not be observed past) the interview date, and were assumed to be independent of censoring.

Second, using the same discrete-time logistic hazard models as Hunter et al. [13], we estimated the marginal proportional hazards (PH; the ratio in probability of sustainment between two levels of a binary factor or a unit change of a continuous predictor) separately for each contextual factor. Missing data in contextual factors were imputed using mean imputation by organizational sustainment status (as in Hunter et al.). We acknowledge there are limitations to using mean imputation, namely that it can artificially reduce variability in a sample; however, we found negligible differences in standard deviations between imputed versus non-imputed factors. Third, we combined data from the replication and original [13] samples and fitted new logistic hazard models including sample (replication vs. original), sample*time (for the survival curve), and factor*sample (for contextual factor analyses) terms to test for between-sample differences. We applied multiple comparison adjustment by types of analyses (i.e., replication sample, combined sample) to control the false discovery rate at the .05 level and avoid excessive false significance [22]. We replicated the cross-sectional models from the original study [13], but also conducted a supplemental analysis adjusting for potential state-level clustering of effects in the replication sample; we found minimal evidence of clustering (see Additional File 5).

#### Perceptions of sustainment factors (qual)

Using rapid qualitative content analysis [23, 24], we summarized perceptions of factors influencing A-CRA sustainment from the provider interviews in the replication sample. A qualitative team member created each summary, and a different team member then coded the summary using a codebook that prioritized factors showing significant quantitative associations with A-CRA sustainment in either sample. We coded until saturation of information was reached, ultimately coding 19 sustaining and 20 non-sustaining organizations (44% of sample). Finally, we developed code summaries for the replication sample that also incorporated qualitative perspectives from the original sample published by Hunter et al. [25]; we did not replicate the methods of that study, because its primarily qualitative approach did not match our QUAN **→** qual design.

## Results

### Sample characteristics

Table 1 shows sustainment rates, by year since grant end, for the replication sample (and the original sample [13] for comparison). The time since grant end ranged from 0–57 months. Participation was not significantly associated with time since grant end (*χ*^2^(4) = 3.55, *p* = .47). Organizations that did not participate had, on average, 0.76 fewer certified clinicians (*p* = .05) and 0.48 fewer certified supervisors (*p* = .01) employed at grant end than participating organizations.

**Table 1:** Estimated survival probability by time since grant end for replication and original samples

Time since grant end (months)	Sample					
	Replication (State-focused grants;* n* = 66)			Original^a^ (Organization-focused grants;* n* = 68)		
	No. of sites observed	No. of sites observed that stopped delivering A-CRA	Estimated survival probability (%)	No. of sites observed	No. of sites observed that stopped delivering A-CRA	Estimated survival probability (%)
0	66	19	71.2	68	9	86.8
1–12	47	5	63.6	42	11	64.0
13–24	36	6	53.0	10	0	64.0
25–36	30	8	38.9	6	2	42.7
37–48	21	0	38.9	1	0	42.7
49–57	7	1	33.3	0	n/a	n/a

*A-CRA* Adolescent Community Reinforcement Approach, *n/a* Not applicable

^a^Data from the original sample were first reported by Hunter et al. [13] and are reported again here to enable comparison to the replication sample

### Probability of A-CRA sustainment

**Fig. 1:**
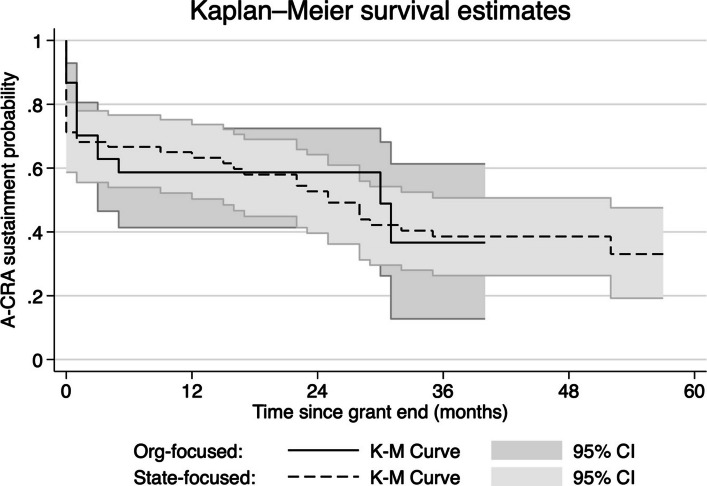
A-CRA = Adolescent Community Reinforcement Approach. K-M = Kaplan–Meier survival analysis. CI = confidence interval. The Kaplan–Meier curve for the original (organization-focused) sample was first reported by Hunter et al. [13] and is reported again here to enable comparison to the replication (state-focused) sample

Figure 1 shows the estimated Kaplan–Meier survival curve for the replication sample (and the original sample [13] for comparison). The survival probabilities (i.e., probability that an organization sustained A-CRA past a given time) were 68.2% (95% confidence interval [CI]: 55.5%, 78.0%) for the first month after grant funding ended and 63.2% (95% CI: 50.3%, 73.7%) after one year. The estimated survival probability steadily trended downward until plateauing at 38.6% (95% CI: 26.3%, 50.7%) by three years and further decreasing to 33.1% (95% CI: 19.2%, 47.6%) by 52 months. Confidence intervals were wider for later years of follow-up because fewer organizations were observed for that long (see Table 1).

Visual inspection suggested that the replication sample and original sample had very similar survival probability curves. This was further confirmed in the combined analysis, where the sample*time term showed no significant effect (PH = 0.06, p = .187, 95% CI: −0.02, 0.15).

### Contextual factors associated with sustainment

Table 2 presents descriptive statistics of contextual factors by A-CRA sustainment status in the replication sample (and the original sample [13] for comparison). Table 3 presents estimated marginal proportional hazards for each contextual factor in the replication sample. Negative PH indicated a lower hazard or higher probability to sustain A-CRA when the factor value was increased, and vice-versa. Table 4 presents combined analyses estimating the main effects of the sample (replication vs. original) and the interaction effects of factor*sample.

**Table 2:** Descriptive statistics for contextual factors by A-CRA sustainment status in the replication and original samples

Contextual factor	Sample			
	Replication (State-focused grants)		Original^a^ (Organization-focused grants)	
	Non-sustainers (*n* = 39)	Sustainers (*n* = 27)	Non-sustainers (*n* = 22)	Sustainers (*n* = 46)
	M (SD) or %	M (SD) or %	M (SD) or %	M (SD) or %
Setting characteristics				
Organizational focus (% substance use)	76.92	96.30	22.73	67.39
No. of services offered	13.26 (2.82)	13.81 (2.06)	11.91 (3.04)	12.24 (2.5)
Communications^b^	3.25 (1.49)	3.72 (1.87)	4.79 (0.89)	4.59 (1.52)
Funding stability^b^	3.01 (0.96)	3.94 (1.88)	2.65 (0.73)	3.82 (1.30)
Organizational capacity^b^	3.79 (1.30)	4.20 (1.49)	5.27 (1.21)	4.95 (1.29)
Partnerships^b^	3.20 (1.55)	3.26 (1.93)	3.98 (1.37)	4.28 (1.35)
Environmental support^b,c^	3.56 (1.32)	4.16 (1.63)	4.37 (1.22)	4.73 (1.19)
Program adaptation^b^	3.84 (1.33)	4.33 (1.56)	5.66 (0.76)	5.25 (0.99)
Program evaluation^b^	3.29 (1.31)	3.96 (1.64)	5.52 (0.85)	5.29 (1.04)
Strategic planning^b^	2.81 (1.12)	3.75 (1.73)	4.07 (1.01)	4.41 (1.37)
Implementation characteristics^d^				
No. of clinicians certified and employed at grant end	0.95 (1.17)	2.48 (1.87)	1.36 (1.26)	2.44 (1.51)
No. of supervisors certified and employed at grant end	0.49 (0.68)	0.93 (0.92)	0.91 (0.87)	0.91 (0.72)
Intervention characteristics				
Complexity	7.9 (2.36)	6.13 (2.61)	7.77 (2.41)	5.78 (1.95)
Implementation difficulty	18.77 (3.21)	17.24 (3.11)	16.27 (3.12)	14.85 (2.84)
Perceived success	16.79 (3.79)	20.06 (2.72)	20.83 (3.90)	20.51 (2.90)
Relative advantage	13.6 (3.13)	14.86 (2.55)	15.86 (3.65)	16.33 (1.93)

*A-CRA* Adolescent Community Reinforcement Approach

^a^Data from the original sample were first reported by Hunter et al. [13] and are reported again here to enable comparison to the replication sample

^b^Hunter et al. [13] reported sum scores for these Program Sustainability Assessment Tool (PSAT) constructs, but we scored the PSAT according to subsequent guidance [18] that recommends calculating an average score for each construct. We rescaled the scores from Hunter et al. so they were comparable; this rescaling does not affect any statistical associations with these variables

^c^Hunter et al. [13] referred to this construct as “political support”

^d^Hunter et al. [13] also examined the number of youth served during the grant period, but data were not available to replicate that analysis with the state-focused sample. This is the only analysis we were unable to replicate

**Table 3:** Logistic regression models estimating the marginal proportional hazards of treatment organizations in the replication sample stopping A-CRA sustainment

Contextual factor	Estimate	SE	Wald CL		Chi-square	Adjusted* p*-value^a^	*p*-value for original sample^b^
			Lower	Upper			
Setting characteristics							
Organizational focus	−0.71	0.39	−1.48	0.06	−1.81	.094^†^	<.001*
No. of services	0.00	0.06	−0.12	0.12	0.03	.975	.45
Communications	−0.11	0.10	−0.31	0.09	−1.09	.316	.52
Funding stability	−0.26	0.11	−0.48	−0.04	−2.30	.042*	<.001*
Organizational capacity	−0.25	0.12	−0.49	0.00	−1.98	.076^†^	.27
Partnerships	−0.04	0.10	−0.23	0.15	−0.38	.751	.87
Environmental support^c^	−0.23	0.11	−0.46	−0.01	−2.08	.067^†^	.03^†^
Program adaptation	−0.17	0.11	−0.39	0.04	−1.57	.143	.14
Program evaluation	−0.27	0.12	−0.50	−0.04	−2.31	.042*	.30
Strategic planning	−0.34	0.12	−0.58	−0.11	−2.82	.015*	.81
Implementation characteristics^d^							
No. of clinicians	−0.73	0.16	−1.05	−0.41	−4.47	.000*	<.001*
No. of supervisors	−0.66	0.25	−1.14	−0.18	−2.67	.020*	.32
Intervention characteristics							
Complexity	0.18	0.06	0.06	0.29	3.05	.009*	<.001*
Implementation difficulty	0.11	0.06	−0.01	0.22	1.85	.093^†^	.02^†^
Perceived success	−0.27	0.05	−0.38	−0.17	−5.26	.000*	.67
Relative advantage	−0.22	0.07	−0.36	−0.08	−3.15	.009*	.11

*n* = 66 treatment organizations

*A-CRA *Adolescent Community Reinforcement Approach*, SE *Standard error*, CL *Confidence limit

^*^Statistical significance at false discovery rate of .05

^†^Statistical significance at false discovery rate of .10

^a^ Reported p-values were adjusted for multiple comparisons using the step-up method [22]

^b^*p*-values from the original sample were first reported by Hunter et al. [13] and are reported again here to enable comparison to the replication sample

^c^Hunter et al. [13] referred to this construct as “political support”

^d^Hunter et al. [13] also examined the number of youth served during the grant period, but data were not available to replicate that analysis with the state-focused sample

**Table 4:** Combined analyses estimating the marginal proportional hazards of the replication versus original samples and interactions with contextual factors

Contextual factor	Estimate for sample term (main effect of sample)	*p*-value	Estimate for sample*factor interaction term	*p*-value
Setting characteristics				
Organizational focus	−0.575	.382	1.240	.155
No. of services	−1.066	.627	0.065	.555
Communications	0.483	.706	−0.190	.474
Funding stability	−2.704	.032*	0.766	.136
Organizational capacity	1.791	.375	−0.457	.155
Partnerships	−0.210	.820	−0.022	.916
Environmental support^a^	−1.626	.375	0.238	.453
Program adaptation	2.270	.375	−0.506	.160
Program evaluation	1.841	.375	−0.484	.155
Strategic planning	0.659	.627	−0.308	.294
Implementation characteristics^b^				
No. of clinicians	−0.992	.283	0.197	.555
No. of supervisors	−0.213	.706	−0.295	.555
Intervention characteristics				
Complexity	1.170	.386	−0.232	.155
Implementation difficulty	1.186	.681	−0.113	.453
Perceived success	3.559	.283	−0.220	.136
Relative advantage	0.374	.820	−0.069	.555

*N* = 134 treatment organizations (*n*s = 66 for replication sample, 68 for original sample [Hunter et al.] [13])

*A-CRA *Adolescent Community Reinforcement Approach

^*^Statistical significance at false discovery rate of .05

^a^Hunter et al. [13] referred to this construct as “political support”

^b^Hunter et al. [13] also examined the number of youth served during the grant period, but data were not available to replicate that analysis with the state-focused sample

At a *p* = .05 false discovery rate, factors significantly associated with A-CRA sustainment in the replication sample (see Table 3) included PSAT ratings of funding stability (PH = −0.26), program evaluation (PH = −0.27), and strategic planning (PH = −0.34); number of A-CRA certified clinicians (PH = − 0.73) and supervisors (PH = − 0.66); and staff perceptions of A-CRA complexity (PH = 0.18), perceived success (PH = −0.27), and relative advantage (PH = −0.22). With a *p* = .10 false discovery rate, four additional factors were significantly related to sustainment: organizational focus (PH = −0.71), organizational capacity (PH = −0.25), environmental support (PH = −0.23) and implementation difficulty (PH = 0.11).

Table 3 also shows that only one significant association from Hunter et al. [13] did not fully replicate: organizational focus was only significant at *p* < .10 (previously *p* < .05). The valence of all PHs was the same across samples. However, several contextual factors uniquely showed significant associations in the replication sample: organizational capacity, program evaluation, strategic planning, number of supervisors, perceived success, and relative advantage.

In the combined analyses (see Table 4), the factor*sample interaction was not significant for any factor. Furthermore, the only significant (*p* < .05) main effect of sample was for funding stability. Finally, the valence of sample main effect and interaction estimates varied considerably (31% positive, 69% negative), with no consistent association between sample and sustainment outcomes. Given the weak evidence for interactions, we relied on the qualitative analyses for further insights rather than attempting to find quantitative explanations.

### Qualitative findings

Several sustainment factors showing statistical associations in the replication sample (see Table 3) were salient in interviews with that sample. Table 5 summarizes how those factors were coded in sustaining versus non-sustaining organizations, with exemplar quotes. Factors with quantitative associations in both the replication and original samples are not presented, as related qualitative findings were also consistent between samples (see Hunter et al. [25]).

**Table 5:** Frequency of discussion in replication sample interviews of contextual factors as barriers and facilitators to A-CRA sustainment by organization sustainment status, with exemplar quotes

Sustainment Factor	Facilitator Summary^a^	Barrier Summary^a^
Organizational capacity	S > NS	S = NS
	*“One thing that our agency does do, is they give educational credit hours, certain allotted days in the year for educational workshops… we could do the A-CRA workshop, we could do any workshop. So it's more something that enhanced someone like myself wanting to even engage and take the time to do the training”* (Facilitator, S interview) *“The trained clinicians all left. So as we'd had the initial group of folks trained under the grant. We had been able to internally train, I think, train an additional one or two folks after that, but then as people left, we weren't able to internally train any more folks”* (Barrier, NS interview)	
Strategic planning	S > NS	S > NS
	*“We were awarded a [grant] that came into effect last year… Part of what we wrote into that grant was the A-CRA program, all staff being trained on that program. All direct service staff being trained in that program in order to implement with the age group that they serve”* (Facilitator, S interview) *“We haven't followed through on [planning to continue A-CRA]… We don't have anything internally”* (Barrier, S interview)	
Program evaluation^b^	N/A	N/A
	N/A	
Number of supervisors	S > NS	S > NS
	*“The supervision I received… one thing about the certification is that… they're pretty rigorous about passing the practice, the audio recording. And it didn't feel pressured, there was flexibility if I was struggling with one that I didn't have to rush through it and get it done. So [my supervisor] was very supportive of that”* (Facilitator, S) *“I think the problem is turnover, so I did have a wonderful A-CRA supervisor who was trying to work with the actual sort of A-CRA people, and our agency to see, is there a way we could develop it or modify it for residential programming? But then there's turnover. So, she went on to another job, and nobody… She was the only A-CRA supervisor for our division, and so it just sort of fell flat after that”* (Barrier, S interview)	
Perceived success	S > NS	N/A
	*“Honestly, I don't have enough good things to say about the program. I will be honest, I implement or I utilize several of the worksheets… so I implement it in practice with pretty much all of my clients at some point, or the ideas that come from that. So I am a huge proponent of the program. I think it's great. It really addresses where the behaviors come from and what is going to perpetuate those behaviors. So I really feel like it meets the adolescents and young adults where they're at mentally, emotionally, cognitively.”* (Facilitator, S interview) N/A for Barrier quotes	
Relative advantage	S > NS	N/A
	*“Number one, our funding source is primarily Medicaid but also private insurance. They want evidence-based practices as much as possible. So we do try to utilize EBPs, including A-CRA, when available”* (Facilitator, NS) N/A for Barrier quotes	

*A-CRA* Adolescent Community Reinforcement Approach, *S* Sustainer organization, *NS *Non-sustainer organization, *N/A* Not applicable (i.e., we did not code any relevant excerpts)

^a^For each sustainment factor, the summary compares number of excerpts coded as facilitators or barriers between S and NS treatment organizations in the replication sample. S > NS indicates that this facilitator or barrier was more salient in sustaining than non-sustaining sites. S < NS indicates less frequently; S = NS indicates an equal amount

^b^Program evaluation was a statistically significant predictor of sustainment in the quantitative results (see Table 3), but participants did not discuss program evaluation in the interviews as a barrier or facilitator. Therefore, qualitative findings (including exemplar quotes) are not available for this factor

Regarding setting characteristics, discussions of organizational capacity in the replication sample focused on available providers (or lack thereof; i.e., turnover) or organizational policies that promoted sustainment, such as having dedicated time and resources for training and flexibility in clinicians’ schedules to deliver A-CRA. Organizational capacity was discussed as a barrier equally by sustaining and non-sustaining organizations, but sustaining organizations noted it as a facilitator more frequently. Although organizational capacity was not a significant predictor in the original sample, those participants’ perceptions of related factors (e.g., internal resources, staff turnover) were similar.

Furthermore, strategic planning was noted as a facilitator by a few sustaining organizations in the replication sample. Such planning led to one organization receiving another grant to train clinicians in A-CRA and another organization creating contracts with state agencies to maintain referrals. In the original sample, planning for long-term A-CRA delivery was described more frequently and at similar levels among sustainers and non-sustainers (likely because the grant mechanism promoted it more [25]).

Regarding implementation characteristics, the number of A-CRA-trained supervisors was a barrier for several non-sustaining organizations in the replication sample. Whether supervisors were never trained in A-CRA or left post-training, absence of a supervisor made it more challenging to train new clinicians in A-CRA. For sustaining organizations in the replication sample, not having enough supervisors was still a barrier, but the presence of a supportive supervisor was more often noted as facilitating sustainment. The number of supervisors was not salient in the original sample interviews.

Finally, regarding intervention characteristics, perceived success and relative advantage were common facilitators for sustaining organizations in the replication sample. Providers viewed A-CRA as effective and attributed its success to A-CRA’s tools, skill-building, strength-based approach, balance between structure and adaptability, and family engagement. The primary relative advantage of A-CRA was its status as an EBP – policies at some state agencies or treatment organizations required delivery of EBP, or even incentivized EBPs through higher reimbursement rates. Non-sustaining organizations in the replication sample discussed the perceived success and relative advantage of A-CRA similarly, but less frequently. Original sample participants described similar A-CRA facilitators, but noted more barriers related to grant-specific requirements (e.g., assessment, data reporting) that they conflated with A-CRA.

## Discussion

We conducted a conceptual replication of Hunter et al.’s [13] study of A-CRA sustainment. We replicated the survival curve for A-CRA sustainment following implementation through federal grants, and we found that sustainment was significantly associated with multilevel contextual factors. Qualitative analyses of interviews suggested that contextual differences, rather than a failure of replication, explain the differences in statistical associations between the two samples.

Our study advances implementation science by conducting a conceptual replication [10], using a large national dataset, and carefully examining interaction effects and qualitative data to guide interpretation of results. The replicated findings help to confirm established theory and empirical work on sustainment drivers [4–8]. Our work may also help inform the design of replication studies by other implementation researchers. The primary limitation was that organizations with fewer certified clinicians or supervisors were less likely to participate, which could limit the generalizability of findings to such organizations.

Unfortunately, our findings suggest that the past decade of research on sustaining youth substance use EBPs has not necessarily produced measurable impacts – which could be the case in many service contexts. Implementation researchers and practitioners can leverage the growing knowledge of EBP sustainment to develop and test robust, multilevel implementation strategies and system-level supports. For example, we are developing and testing sustainable A-CRA training strategies that may decrease ongoing training and implementation burdens to better align with typical organizational and funding capacities; examples include artificial-intelligence-based strategies that streamline training (e.g., virtual composite clients and supervisees for training, automated model fidelity review of trainee session content) and training school-based providers to promote accessibility and continuity of A-CRA services. Research can also draw on recent advances in conceptualizing sustainment strategies [26, 27] and outcome measures [28, 29]. Such efforts can help to improve, rather than replicate, sustainment trajectories over the next decade.

## Supplementary Information

Additional file 1.

Additional file 2.

Additional file 3.

Additional file 4.

Additional file 5.

## Data Availability

The datasets analyzed for this study are available from the corresponding author on reasonable request. Depending on the nature of the request, institutional data-sharing agreements may or may not be required.

## Abbreviations

A-CRA: Adolescent Community Reinforcement Approach
CFIR: Consolidated Framework for Implementation Research
CHS: Chestnut Health Systems
EBP: Evidence-based practice
SAMHSA: Substance Abuse and Mental Health Services Administration
SUD: Substance use disorder
