# Supplementary material for: Minimal progress toward sustainment: 10-year replication of substance use EBP sustainment trajectories and associations with implementation characteristics
Source: Implement Sci. 2025 Dec 2;21:3. doi: 10.1186/s13012-025-01471-2 (PMC12777219; doi:10.1186/s13012-025-01471-2)
Supplement: Supplementary file 2 — Additional file 2. [file 13012_2025_1471_MOESM2_ESM.docx]

**Additional File 2. Overview of Original Study (Hunter et al., 2015)**

This file provides an overview of the sample, methods, and results of Hunter et al. (2015; *Implementation Science*)[13], which examined sustainment of the Adolescent Community Reinforcement Approach (A-CRA) for youth substance use in cohorts of treatment organizations. This overview provides important context for the present study, which was a conceptual replication of Hunter et al. conducted with more recent cohorts of treatment organizations.

*Sample*

The Hunter et al. (2015)[13] original study sample included data from 68 treatment organizations operating across 27 states that received discretionary grant funding from the U.S. Substance Abuse and Mental Health Services Administration (SAMHSA) to support implementation of A-CRA, awarded between 2006-2010. That study recruited staff from treatment organizations by email or phone to participate in interviews and surveys, with the goal to include both clinical supervisors and clinicians from each organization who had knowledge about A-CRA. Of the 82 treatment organizations eligible to participate, 68 had staff participate (83% response rate). We used the same recruitment strategies for the current replication study with new cohorts of SAMHSA grantees.

A key difference between the two studies is that the cohorts of treatment organizations included in each sample had implemented A-CRA through different types of federal grants, making the current study a *conceptual* replication. In Hunter et al.’s (2015)[13] original (i.e., “organization-focused”) sample, the funded treatment organizations (a) applied for and received funding directly from SAMHSA with no involvement from state substance use agencies and (b) were responsible for grant activities and reporting to SAMHSA. In contrast, in the present replication study (i.e., “state-focused”) sample, state substance use service agencies applied for and were responsible for grant activities and reporting to SAMHSA; those agencies then worked with treatment organizations in their state to implement A-CRA.

*Data Collection*

Hunter et al. (2015)[13] originally collected data from the organization-focused sample through semi-structured interviews tailored to the provider’s role and whether the organization still delivered A-CRA, followed by a web-based survey that collected standardized measures and other descriptive information. The replication study used similar interview and survey protocols conducted with the state-focused sample; those protocols had some modifications (e.g., we added questions about impacts of COVID-19 pandemic), but we only used data collected consistently across both samples for the replication analyses. Additional Files 3 and 4 provide copies of the interview and survey protocols, respectively, used with each sample to enable direct comparisons. Hunter et al. also collected administrative data on the number of youth treated with A-CRA during the grant period for each treatment organization; those data were not available for the state-focused sample, so we could not replicate that analysis in the current study (this is the only analysis we were unable to replicate).

At the time of planning the original Hunter et al. (2015)[13] study, there was little empirical evidence to suggest which factors would best predict sustainment of evidence-based treatments for youth substance use. Therefore, the authors took an exploratory approach that examined 17 contextual factors (setting, sustainability planning, implementation characteristics, and intervention characteristics) potentially associated with sustainment. The Program Sustainability Assessment Tool (PSAT)[17,18] was one of the few measurement tools available to assess the specific constructs conceptualized to be related to program sustainment. The Consolidated Framework of Implementation Research (CFIR)[4] was also newly available, but it provides domains and constructs theorized to be associated with implementation, not sustainment. Sustainment, however, cannot be possible without first achieving successful implementation, so Hunter et al. determined that assessing contextual factors from both PSAT and CFIR was ideal.[17,18,19,20] (Of note, at that time CFIR included 39 constructs in 5 domains; they did not include all elements).

*Data Analysis*

The first analytic step in Hunter et al. (2015)[13] was to calculate a discrete-time survival analyses using the staff-reported termination date for A-CRA as the event occurrence. More specifically, a Kaplan-Meier survival curve was fitted using time-to-event as the number of months between the end of the SAMHSA grant and the time the organization stopped delivering A-CRA. Sustainment outcomes were right censored by (i.e., could not be observed past) the interview date, and were assumed to be independent of censoring. In the current study, we replicated this analytic step using the state-focused sample and then directly compared the survival curves generated from the two samples.

Next, Hunter et al. (2015)[13] examined the 17 factors potentially associated with A-CRA sustainment status using discrete-time logistic hazard models to estimate the marginal proportional hazards for each contextual factor (i.e., the ratio in probability of sustainment between two levels of a binary factor or a unit change of a continuous predictor). To control the false discovery rate for analyses at the .05 level, they applied multiple comparison adjustment to *p*-values[22]. Hunter et al. also used several approaches for handling missing data, including mean imputation, multiple imputation, and complete-case analysis. Their main results were based on mean imputation, noting that the alternative approaches for handling missing data had produced similar results. In the current study, we followed suit by reporting main results based on mean imputation for the purpose of replication, and we used the same multiple comparison adjustment. We replicated marginal proportional hazard models for 16 of the 17 factors (as noted previously, number of youth treated could not be tested in the replication study) and then combined the original and replication sample for analyses that included sample, sample*time, and factor*sample terms for more direct comparison of findings by sample.

*Results*

When data were collected from the original sample for Hunter et al. (2015)[13] in Fall 2013, the treatment organizations varied in the time since their SAMHSA grant ended, from approximately 1 to 48 months. Participation was not significantly associated with time since grant end (χ^2^(4)=3.53, *p*=0.53), and there were no significant differences between organizations that participated and those that did not participate on implementation variables (i.e., the number of youth treated, the number of certified staff employed at grant end). In the current manuscript, Table 1 shows sustainment rates, by year since grant funding ended, for both the original sample (for comparison, taken directly from Hunter et al.) and the replication sample.

Hunter et al. (2015)[13] reported that in the original sample, the survival probabilities (i.e., probability that an organization sustained A-CRA past a given time) were 86.8% (95% confidence interval [CI]: 79.1%, 95.2%) for the first month after the grant funding ended and 58.7% (95% CI: 44.8 %, 76.7%) after one year. The estimated survival probability then remained flat until month 32, where it dropped to 37% (95% CI: 17.8%, 75.4%). However, the 95% CIs were wide at month 32 because fewer organizations were observed for that long. In the current manuscript, Figure 1 shows the estimated Kaplan-Meier survival curves for both the original sample (for comparison, taken directly from Hunter et al.) and the replication sample.

Controlling the false discovery rate at .05, factors significantly associated with A-CRA sustainment in the original sample (Hunter et al., 2015[13]) included: organizational focus (PH = -1.90), funding stability (PH = -0.20), number of clinicians certified at grant end (PH = -0.88), and intervention complexity (PH = 0.41). A negative PH indicated a lower hazard or higher probability to sustain A-CRA when the factor value increased, and vice-versa. With a *p*=.10 false discovery rate, three additional factors were significantly related to sustainment: political support (PH = -0.09), number of youth treated with A-CRA during the grant period (PH = -0.01), and intervention difficulty (PH = 0.21). (Note that the factor called “political support” in Hunter et al. is now referred to as “environmental support” throughout the current manuscript.) In other words, Hunter et al. found that the probability of sustaining A-CRA was greater in organizations that: were primarily substance use treatment-oriented; reported higher rates of funding stability to support A-CRA delivery; trained and retained more A-CRA certified clinicians; treated more youth during the funding period; and rated A-CRA as less complex and less difficult to implement.

In the current manuscript, Table 2 presents descriptive statistics of contextual factors by A-CRA sustainment status for both the original sample (for comparison, taken from Hunter et al., 2015[13]) and the replication sample. Note that Hunter et al. originally reported sum scores for PSAT constructs, but in this manuscript, we scored the PSAT according to subsequent guidance[18] that recommends calculating an average score for each construct. To permit direct comparisons, the values from Hunter et al. reported in Table 2 have been rescaled to average scores; this rescaling does not affect any statistical associations with these variables. Table 3 in the current manuscript reports the estimated marginal proportional hazards for contextual factors in both the original (for comparison, taken directly from Hunter et al.) and replication samples.
