# Supplementary material for: Minimal progress toward sustainment: 10-year replication of substance use EBP sustainment trajectories and associations with implementation characteristics
Source: Implement Sci. 2025 Dec 2;21:3. doi: 10.1186/s13012-025-01471-2 (PMC12777219; doi:10.1186/s13012-025-01471-2)
Supplement: Supplementary file 5 — Additional file 5. [file 13012_2025_1471_MOESM5_ESM.docx]

**Supplemental analysis: Impact of state-level clustering in marginal proportional hazard models for associations between contextual factors and sustainment of the Adolescent Community Reinforcement Approach (A-CRA)**

Given that our replication sample came from cohorts of grants that were awarded to state substance use service agencies, state-level clustering is an understandable concern when modeling effects with that sample. For replication purposes, we needed to apply the same modeling specification as the original study with grants awarded directly to treatment organizations (Hunter et al., 2015); that study adopted cross-sectional modeling assumption and did not adjust for state-level clustering. In a supplemental analysis presented here, we explored the impact of state-level clustering given the different context of the replication sample.

We first examined the empirical distribution of state-level clusters in the replication sample. There were 18 state clusters with a range of 2-5 treatment organizations per state; the most common cluster size was 3 organizations per state. Next, we refitted the discrete-time survival analysis models using the Huber-White robust standard error to adjust for potential clustering at the state level. This technique does not affect point estimates from the survival analysis models, but it produces adjusted standard errors that account for clustering; on average, it is expected the standard error will increase and the statistical power will be weakened when accounting for clustering. We compiled the comparison in statistical inferences, i.e., adjusted p-values, between our primary analysis (presented in Table 3) and the alternative adjusted *p*-values with state-clustered standard errors. Table S1 summarizes the results and notes differences.

Table S1.

| Contextual Factor | Adjusted *p*-value from Table 3 (primary analysis) | Alternative adjusted *p*-value with state-clustered SE | Change in significance level when using the state-clustered SE |
| --- | --- | --- | --- |
| Organizational focus | 0.094^†^ | 0.051^†^ | No change |
| No. of services offered | 0.975 | 0.974 | No change |
| Communications | 0.316 | 0.237 | No change |
| Funding stability | 0.042* | 0.009* | No change |
| Organizational capacity | 0.076^†^ | 0.061^†^ | No change |
| Partnerships | 0.751 | 0.711 | No change |
| Environmental support | 0.067^†^ | 0.024* | Increase, .1 to .05 |
| Program adaptation | 0.143 | 0.061^†^ | Increase, none to .1 |
| Program evaluation | 0.042* | 0.061^†^ | Decrease, .05 to .1 |
| Strategic planning | 0.015* | 0.011* | No change |
| No. of clinicians | 0.000* | 0.015* | No change |
| No. of supervisors | 0.020* | 0.051^†^ | Decrease, .05 to .1 |
| Complexity | 0.009* | 0.061^†^ | Decrease, .05 to .1 |
| Implementation difficulty | 0.093^†^ | 0.124 | Decrease, .1 to none |
| Perceived success | 0.000* | 0.009* | No change |
| Relative advantage | 0.009* | 0.017* | No change |

SE = standard error.

*Statistical significance at false discovery rate of .05.

^†^Statistical significance at false discovery rate of .10.

Overall, this supplemental analysis does not demonstrate a notable or consistent impact of state-level clustering in our findings. As expected, we saw a small overall decrease in power; the significance level decreased for four factors, whereas the significance level increased for two factors. Overall, these findings did not suggest the resulting decrease in power to conduct analyses with state-level clustering was warranted – especially since it does not replicate the prior study – and therefore we did not incorporate adjustment for state-level clustering into the main analyses for our study.
